# Vascular Endothelial Growth Factor Inhibitors and the Risk of Aortic Aneurysm and Aortic Dissection

**DOI:** 10.1001/jamanetworkopen.2024.0940

**Published:** 2024-03-04

**Authors:** Chia-Wei Wu, Hsin-Yi Huang, Shin-Yi Lin, Chi-Chuan Wang, Chih-Fen Huang, I-Hui Wu

**Affiliations:** 1 Department of Pharmacy, National Taiwan University Hospital, Taipei, Taiwan; 2 School of Pharmacy, College of Medicine, National Taiwan University, Taipei, Taiwan; 3 Graduate Institute of Clinical Pharmacy, College of Medicine, National Taiwan University, Taipei, Taiwan; 4 Department of Traumatology, National Taiwan University Hospital, Taipei, Taiwan; 5 Department of Cardiovascular Surgery, National Taiwan University Hospital, Taipei, Taiwan; 6 Graduate Institute of Clinical Medicine, College of Medicine, National Taiwan University, Taipei, Taiwan

## Abstract

**Importance:**

Vascular endothelial growth factor pathway inhibitors (VPIs) pose a concern for aortic aneurysm (AA) and aortic dissection (AD), signaling potential vascular disease development.

**Objective:**

To investigate VPI-associated AA and AD.

**Design, Setting, and Participants:**

This case-control study with a nested design used full population data from a national claims database in Taiwan between 2011 and 2019. Eligible participants were aged 20 years or older with kidney, hepatic, gastrointestinal, or pancreatic cancer diagnosed between January 1, 2012, and December 31, 2019. The first cancer diagnosis date was defined as the cohort entry date. Cases were patients who received a diagnosis of AA or AD in hospitalizations or emergency visits between the cohort entry date and December 31, 2019. Controls were matched by ratio (up to 1:5) based on age, sex, cancer type, cohort entry date, and the index date (ie, the first AA or AD event date). Data analysis was performed between January 2022 and December 2023.

**Exposures:**

Use of the oral VPIs sorafenib, sunitinib, and pazopanib between cohort entry date and index date.

**Main Outcomes and Measures:**

In the primary analysis, AA and AD were evaluated compositely, while in the secondary analyses, they were evaluated separately. Adjusted odds ratios (aORs) were calculated using conditional logistic regression to assess the association with VPI use (sorafenib, sunitinib, and pazopanib) considering various VPI exposure windows and cumulative use.

**Results:**

A total of 1461 cases were included (mean [SD] age, 73.0 [12.3] years; 1118 male patients [76.5%]), matched to 7198 controls. AA or AD risk increased with a VPI exposure of 100 days or less before the index date (aOR, 2.10; 95% CI, 1.40-3.15), mainly from VPI-associated AD (aOR, 3.09; 95% CI, 1.77-5.39). Longer VPI duration (68 days or more: aOR, 2.64; 95% CI, 1.66-4.19) and higher cumulative dose (61 or more defined daily doses: aOR, 2.65; 95% CI, 1.66-4.23) increased the risk.

**Conclusions and Relevance:**

The use of the 3 study VPIs (sorafenib, sunitinib, and pazopanib) was associated with an increased risk of AA and AD in patients with cancer, essentially all of the risk from VPI-associated AD. Future studies are needed to determine the risk factors of VPI-associated AA and AD, as well as to establish a class effect.

## Key Points

**Question:**

Is the use of vascular endothelial growth factor pathway inhibitors associated with the risk of aortic aneurysm and aortic dissection in patients with cancer?

**Findings:**

This case-control study of 8659 individuals found that the use of sorafenib, sunitinib, and pazopanib was significantly associated with a 2-fold increase in the risk of aortic aneurysm and aortic dissection in patients with cancer. Essentially all of the risk arose from aortic dissection events.

**Meaning:**

These results suggest that patients using vascular endothelial growth factor pathway inhibitors should be monitored carefully for the potential risk of aortic aneurysm and aortic dissection.

## Teaser

This case-control study of Taiwanese adults diagnosed with cancer examines the association of the use of vascular endothelial growth factor pathway inhibitors and risk of aortic aneurysm and aortic dissection.

## Introduction

The vascular endothelial growth factor (VEGF) pathway has several therapeutic targets for tumor angiogenesis.^1,2^ In 2004, the US Food and Drug Administration (FDA) approved VEGF pathway inhibitors (VPIs) for the treatment of malignant neoplasms such as hepatocellular carcinoma and renal cell carcinoma, as well as their metastases.^3^

As VEGF pathway inhibition may lead to endothelial dysfunction or impaired vessel wall integrity, VPI-associated cardiovascular toxicities is of concern.^2,3,4,5,6^ Recent pharmacovigilance database analyses have reported a 2.8- to 22.3-fold increase in the reporting rate of aortic aneurysm (AA) and aortic dissection (AD) associated with VPI use,^7,8,9,10,11^ with a hospitalization rate of 29.8% and mortality rates ranging from 19.9% to 24.3%.^7,10,11^ In 2019 and 2020, medicines agencies in European and the UK issued safety alerts to health care professionals regarding VPI-associated AA and AD.^12,13^ In addition, the FDA is currently evaluating whether regulatory action is required for VPI-associated AA and AD.^14^ However, to date, no randomized clinical trials and only limited population-based studies have been conducted to examine the relationship between VPI use and the development of AA and AD.^7,8,9,10,11,15^ The aim of this study was to investigate the association between VPI use and the risk of AA and AD in patients with cancer using data from a nationwide claims database.

## Methods

This study was granted an exemption from institutional review board review by the Research Ethics Committee of the National Taiwan University Hospital—the study did not require informed consent because only deidentified information was used. The study followed the Strengthening the Reporting of Observational Studies in Epidemiology (STROBE) reporting guideline for case-control studies.

### Data Source

This study was conducted with a nationwide claims database. Two data sources were used for this study: the 2011-2019 full population data sets from the National Health Insurance Research Database (NHIRD) and Taiwan Cancer Registry (TCR).^16^ Further information on the data source is available in eMethods in Supplement 1.

### Study Design

This study employed a nested case-control study design using the NHIRD data from January 1, 2011, to December 31, 2019. We chose the nested case-control design because of the relatively low average incidence of AA and AD.^17,18^ Data analysis was performed between January 2022 and December 2023. Detailed information on study design is available in eMethods in Supplement 1.

### Study Patients

This study included patients who were diagnosed with kidney, hepatic, gastrointestinal, or pancreatic cancer, which were indications reimbursed by the NHI program for the study VPIs (eTable 1 in Supplement 1), between January 1, 2012, and December 31, 2019. Patients were considered to have cancer if they had received at least 1 inpatient diagnosis or 2 outpatient diagnoses for our specified cancer types. The date of the first cancer diagnosis within the study period was defined as the cohort entry date. Patients at least 20 years old at the cohort entry date were included. Patients who had a history of AA or AD or had been prescribed VPIs between January 1, 2011, and the cohort entry date were excluded. The *International Classification of Diseases, Ninth Revision, Clinical Modification* (*ICD-9-CM*) and *ICD, Tenth Revision, Clinical Modification* (*ICD-10-CM*) diagnosis codes were used to define cancer and other medical conditions (eTable 2 in Supplement 1).

Given that VPIs can be prescribed to patients with late-stage cancer or those who have failed previous cancer treatment under the NHI reimbursement scheme, we included all patients with kidney, hepatic, gastrointestinal, or pancreatic cancer regardless of their initial stage in the main analysis to maximize the sample size (eTable 1 in Supplement 1). This approach allowed us to include all potential VPI users, including those who were diagnosed with advanced stage and those who progressed to the advanced stages and thus received VPIs. We further conducted a subgroup analysis by linking the TCR to the NHIRD to identify patients diagnosed with stage IV or metastatic cancer at the time of their initial diagnosis.

### Cases and Controls

Cases were defined as patients who were hospitalized or had visited the emergency department (ED) with a primary diagnosis of AA or AD between the cohort entry date and December 31, 2019. The index date for the cases was defined as the date of their first AA or AD event. To match cases, controls were selected from the same patient pool at a ratio of up to 1:5 based on age (within 2 years), sex, cancer type, and cohort entry date (within 90 days) using risk sets from patients who had not experienced an AA or AD event at the time a case occurred. Consistent with the cases, the index date of the controls was defined by a hospitalization or an ED visit not related to AA or AD that occurred as the same date as the index date for a case. This approach ensured a similar time period between the cohort entry date and the index date for cases and matched controls. By employing the risk set sampling approach to choose controls, individuals who later became cases were allowed to be considered as eligible controls before they developed the event of interest. AA and AD were defined using *ICD-9-CM* code 441 and *ICD-10-CM* codes I71, I79.0, and I79.1,^19,20^ which have been validated in a previous study (eTable 3 in Supplement 1).^19^

### VPI Exposure

The exposure of interest in this study was 3 oral VPIs, sorafenib, sunitinib, and pazopanib, which have been reimbursed by the NHI program since before 2012 (eTable 1 in Supplement 1).^21,22^ The Anatomical Therapeutic Chemical (ATC) codes used to identify the VPIs and other medications are listed in eTable 4 in Supplement 1.

The risk exposure period was defined as the period between the cohort entry date and the index date. Patients who used VPIs during the risk exposure period were considered exposed, while those who did not use VPIs were considered unexposed. Because the onset of VPI-associated AA and AD varied according to previous studies,^8,9,10,11,15^ we then divided the risk exposure periods into 3 risk windows to further explore whether the timing of exposure to VPIs would influence the risk of AA and AD. These risk windows included within 100 days before the index date, 101 to 365 days before the index date, and more than 365 days before the index date. These 3 risk windows were determined based on previous studies, which reported a median time to onset of between 79.5 and 114 days and a 75th percentile time to onset of 212 to 393 days.^8,9,10,11,15^ Patients were considered exposed to VPIs in a given risk window if their last VPI prescription ended in that window.

The risk of AA and AD was also evaluated on the basis of the cumulative duration and cumulative dose of VPI use during the risk exposure period. Given the diverse onset times associated with VPI-related AA and AD and the absence of evidence indicating a dose-dependent effect,^8,9,10,11,15^ defining a specific cut-off point for an elevated risk of AA and AD based on cumulative duration or dose was challenging. Consequently, we adopted the median cumulative duration or dose as the reference point to stratify patients into 2 groups. Further information on VPI exposure is available in eMethods in Supplement 1.

### Covariates

Covariates adjusted in this study were age, sex, cohort entry year, cohort entry season, cancer type, Charlson Comorbidity Index,^23^ comorbidities, and concomitant medications. Comorbidities associated with the risk of AA and AD were identified between January 1, 2011, and the cohort entry date (eTable 2 in Supplement 1).^19,20^ Adjustments were made for concomitant medications prescribed within 365 days before the cohort entry date as baseline drugs and within 100 days prior to the index date as current co-exposure drugs (eTable 4 in Supplement 1).^19,20^ To ensure the medications were used for long-term treatment, only those prescribed continuously for more than 7 days were documented.

### Statistical Analysis

To evaluate the differences between the case and control groups in baseline covariates, categorical variables were expressed as numbers and percentages and compared using a Fisher exact test or the χ^2^ test. Continuous variables were reported as mean averages and compared using either a Student *t* test or Mann-Whitney U test. To evaluate the association of VPI use with AA and AD, conditional logistic regression models were used. We calculated unadjusted odds ratios (ORs) and adjusted ORs (aOR). Covariates that showed significant differences between the case and control groups were included in the regression model to calculate the aORs. These covariates were considered clinically relevant as potential risk factors or confounders to AA and AD. Two sets of analyses were performed. In the primary analysis, the risk of AA and AD was evaluated as a composite event, while in the secondary analyses, the risk of AA and AD was evaluated as 2 separate events. The statistical significance threshold was set at *P* < .05 in 2-sided tests. All statistical analyses were performed using SAS version 9.4 (SAS Institute).

## Results

We began with 424 253 patients with kidney, hepatic, gastrointestinal, or pancreatic patients with cancer in the NHIRD (Figure). After applying the exclusion criteria, we identified 417 302 eligible patients for analysis. From this patient pool, 1461 cases of AA and AD were identified, consisting of 343 (23.5%) female and 1118 (76.5%) male patients; 184 (12.6%) patients were diagnosed with kidney cancer, 444 (30.4%) with hepatic cancer, 696 (47.6%) with gastrointestinal cancer, and 114 (7.8%) with pancreatic patients with cancer (Table 1). The mean (SD) age of these cases was 73.0 (12.3) years. These cases were then matched to 7198 controls. Further information on patient comorbidities and concomitant medications is available in eTable 5 in Supplement 1.

**Figure.**
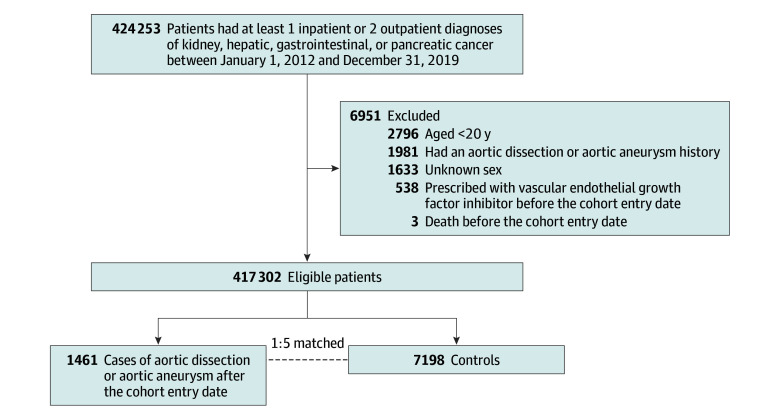
Selection Process of the Cases and Controls

**Table 1.** Patient Demographics

Demographics	Patients, No. (%)	
	Cases (n = 1461)	Controls (n = 7198)
Sex		
Female	343 (23.5)	1670 (23.2)
Male	1118 (76.5)	5528 (76.8)
Age, mean (SD), y	73.0 (12.3)	72.7 (12.2)
Time between cohort entry date and index date, median (IQR), mo	10.5 (0.2-39.8)	10.7 (2.1-40.4)
Index year		
2012	130 (8.9)	622 (8.6)
2013	156 (10.7)	757 (10.5)
2014	157 (10.6)	779 (10.8)
2015	180 (12.3)	884 (12.3)
2016	176 (12.1)	870 (12.1)
2017	201 (13.8)	997 (13.9)
2018	252 (17.3)	1254 (17.4)
2019	209 (14.3)	1035 (14.4)
Index season		
December-February	373 (25.5)	1841 (25.6)
March-May	367 (25.1)	1816 (25.2)
June-August	352 (24.1)	1730 (24.0)
September-November	369 (25.3)	1811 (25.2)
Cancer type		
Kidney cancer	184 (12.6)	919 (12.8)
Hepatic cancer	444 (30.4)	2208 (30.7)
Gastrointestinal cancer	696 (47.6)	3471 (48.2)
Pancreatic cancer	114 (7.8)	547 (7.6)
≥2 Types of cancer	23 (1.6)	53 (0.7)

In the primary analysis, we identified 49 patients exposed to VPI in the case group and 133 patients exposed to VPI in the control group (Table 2). The proportions of AA and AD were 26.9% (49 of 182 patients) in the VPI exposure group and 16.7% (1412 of 8477 patients) in the nonexposure group. The unadjusted analysis indicated that VPI use was associated with an increased risk of AA and AD (unadjusted OR, 1.93; 95% CI, 1.37-2.72). The association between VPI use and the risk of AA and AD persisted after adjusting for baseline differences (aOR, 2.00; 95% CI, 1.41-2.84). The secondary analyses revealed the increased risk was essentially from VPI-associated AD (aOR, 3.09; 95% CI, 1.77-5.39), while a nonsignificant increase in the risk of AA associated with VPI use was observed (aOR, 1.49; 95% CI, 0.91-2.45).

**Table 2.** Association Between VPI Use and the Risk of AA and AD^a^

Characteristic	Patients, No. (%)		OR (95% CI)	
	Cases, No. (%)	Controls, No. (%)	Unadjusted	Adjusted^b^
**Primary analysis**				
AA or AD^c^				
Total, No.	1461	7198	NA	NA
VPI exposure	49 (26.9)	133 (73.1)	1.93 (1.37-2.72)	2.00 (1.41-2.84)
Nonexposure	1412 (16.7)	7065 (83.3)	1 [Reference]	1 [Reference]
**Secondary analyses**				
AA only^c^				
Total, No.	955	4686		
VPI exposure	22 (21.8)	79 (78.2)	1.41 (0.87-2.30)	1.49 (0.91-2.45)
Nonexposure	933 (16.8)	4607 (83.2)	1 [Reference]	1 [Reference]
AD only^c^				
Total, No.	443	2197	NA	NA
VPI exposure	24 (34.3)	46 (65.7)	2.91 (1.71-4.96)	3.09 (1.77-5.39)
Nonexposure	419 (16.3)	2151 (83.7)	1 [Reference]	1 [Reference]

Abbreviations: AA, aortic aneurysm; AD, aortic dissection; NA, not applicable; OR, odds ratio; VPI, vascular endothelial growth factor pathway inhibitor.

^a^ VPIs included sorafenib, sunitinib, and pazopanib.

^b^ Adjusted ORs were obtained using multivariable conditional logistic regression models. Baseline conditions with statistically significant OR differences between cases and controls were adjusted as covariates; these included cerebrovascular disease, coronary artery disease, peripheral arterial disease, diabetes, hypertension, aortic valve disease, chronic obstructive pulmonary disease, asthma, chronic kidney disease, tobacco use, Charlson Comorbidity Index, concomitant medication use within 365 days before the cohort entry date (oral hypoglycemic agents and nitrates), and concomitant medication use within 100 days before the index date (statins, antiarrhythmic agents, digoxin, and insulin) for the outcome of AA or AD; eTables 6 and 7 in Supplement 1 present the covariates in the multivariable conditional logistic regression models for the outcomes of AA only and AD only.

^c^ Overall risk exposure: between the cohort entry date (ie, the date of the initial cancer diagnosis) and the index date (ie, the date of the first AA or AD event).

Regarding the association between VPI use and the risk of AA and AD across different risk windows, we observed an increased risk of AA and AD among patients exposed to VPIs within the 100 days before the index date compared with patients who were unexposed to VPIs (aOR, 2.10; 95% CI, 1.40-3.15) (Table 3). However, there was no significant increase in the risk of AA and AD among patients exposed to VPIs between 101 and 365 days and over 365 days before the index date compared with unexposed patients.

**Table 3.** Association Between VPIs Use and the Risk of AA and AD Across Different Risk Windows^a^

Risk windows of VPI exposure (time before index date)^b^	Patients, No. (%)		OR (95% CI)	
	Cases (n = 1461)	Controls (n = 7198)	Unadjusted	Adjusted^c^
≤100 d	37 (27.6)	97 (72.4)	2.01 (1.35-3.00)	2.10 (1.40-3.15)
101-365 d	5 (23.8)	16 (76.2)	1.57 (0.57-4.35)	1.66 (0.60-4.61)
>365 d	7 (25.9)	20 (74.1)	1.83 (0.76-4.40)	1.87 (0.77-4.51)
Nonexposure	1412 (16.7)	7065 (83.3)	1 [Reference]	1 [Reference]

Abbreviations: AA, aortic aneurysm; AD, aortic dissection; OR, odds ratio; VPI, vascular endothelial growth factor pathway inhibitor.

^a^ VPIs included sorafenib, sunitinib, and pazopanib.

^b^ The date of the first AA or AD event.

^c^ Adjusted ORs were obtained using multivariable conditional logistic regression models. Baseline conditions with statistically significant OR differences between cases and controls were adjusted as covariates; these included cerebrovascular disease, coronary artery disease, peripheral arterial disease, diabetes, hypertension, aortic valve disease, chronic obstructive pulmonary disease, asthma, chronic kidney disease, tobacco use, Charlson Comorbidity Index, concomitant medication use within 365 days before the cohort entry date (oral hypoglycemic agents and nitrates), and concomitant medication use within 100 days before the index date (statins, antiarrhythmic agents, digoxin, and insulin).

According to the distribution of VPI prescriptions among cases with AA and AD and matched controls, the median cumulative duration was 68 days, and the median cumulative dose was 61 defined daily dose (DDD). The median values of cumulative duration and dose were used as the cut-off points to evaluate the dose-dependent correlation of VPI-associated AA and AD. When considering the cumulative use of VPIs, a duration of 68 days or more was associated with an increased risk of AA and AD (aOR, 2.64; 95% CI, 1.66-4.19) (Table 4). In addition, a VPI cumulative dose of 61 DDDs or more was associated with an increased risk of AA and AD (aOR, 2.65; 95% CI, 1.66-4.23). The same findings were observed in the secondary analyses for VPI-associated AD, and similar trends were seen between VPI use and the risk of AA (eTables 6 and 7 in Supplement 1).

**Table 4.** Association Between Cumulative VPIs Use and the Risk of Aortic Aneurysm and Aortic Dissection^a^

Characteristic	Patients, No. (%)		OR (95% CI)	
	Cases (n = 1461)	Controls (n = 7198)	Unadjusted	Adjusted^b^
**VPI cumulative duration** ^c^				
<68 d	19 (21.1)	71 (78.9)	1.39 (0.83-2.33)	1.45 (0.86-2.47)
≥68 d	30 (32.6)	62 (67.4)	2.57 (1.63-4.03)	2.64 (1.66-4.19)
Nonexposure	1412 (16.7)	7065 (83.3)	1 [Reference]	1 [Reference]
**VPI cumulative doses** ^c^				
<61 DDDs^d^	20 (22.2)	70 (77.8)	1.47 (0.88-2.46)	1.47 (0.88-2.48)
≥61 DDDs^d^	29 (31.5)	63 (68.5)	2.46 (1.55-3.89)	2.65 (1.66-4.23)
Nonexposure	1412 (16.7)	7065 (83.3)	1 [Reference]	1 [Reference]

Abbreviations: DDD, defined daily dose; OR, odds ratio; VPI, vascular endothelial growth factor pathway inhibitor.

^a^ VPIs included sorafenib, sunitinib, and pazopanib.

^b^ Adjusted ORs were obtained using multivariable conditional logistic regression models. Baseline conditions with statistically significant OR differences between cases and controls were adjusted as covariates; these included cerebrovascular disease, coronary artery disease, peripheral arterial disease, diabetes, hypertension, aortic valve disease, chronic obstructive pulmonary disease, asthma, chronic kidney disease, tobacco use, Charlson Comorbidity Index, concomitant medication use within 365 days before the cohort entry date (oral hypoglycemic agents and nitrates), and concomitant medication use within 100 days before the index date (statins, antiarrhythmic agents, digoxin, and insulin).

^c^ Between the cohort entry date (ie, the date of the initial cancer diagnosis) and the index date (ie, the date of the first aortic aneurysm or aortic dissection event).

^d^ According to the World Health Organization, the DDDs were 800 mg for sorafenib, 33 mg for sunitinib, and 800 mg for pazopanib.

In the subgroup analysis for advanced patients with cancer, we observed a nonsignificant increased risk of AA and AD (unadjusted OR, 2.00; 95% CI, 0.18-22.06) among patients exposed to VPIs within the 100 days before the index date. However, we were unable to conduct adjusted analysis due to the limited sample size (eTable 8 in Supplement 1).

## Discussion

This study was, to the best of our knowledge, the first nested case-control study to assess the association between VPI exposure and risk of AA and AD in real-world settings using a nationwide database. The results of this study demonstrated that the use of sorafenib, sunitinib, and pazopanib was associated with a 2-fold increase in the risk of AA and AD in patients with cancer. This finding was consistent with a 2023 cohort study using national claims data from Korea.^15^ Additionally, our study further indicated that all of the risk of VPI-associated AA and AD was essentially driven by AD events.

Previous studies have indicated a median onset time of 79.5 to 114 days for VPI-associated AA and AD.^7,8,9,10,11,15^ In our study, current exposure to VPIs, particularly within the 100 days before the index date, was significantly associated with a higher risk of AA and AD compared with nonexposure.

Generally, the mechanism underlying VPI-associated AA and AD remains unclear. VPIs interrupt VEGF pathway signaling and consequently reduce the production of nitric oxide and activate the endogenous endothelin system. This may result in de novo hypertension in 30% to 80% of patients after VPI therapy.^2,4,5,6,8^ Elevated blood pressure caused by preexisting hypertension or inhibition of the VEGF pathway may contribute to the development of AA and AD.^24,25^ Previous studies have found that the risk of VPI-associated AD was similar in patients with and without preexisting hypertension.^8,9^ Our study also indicated that the risk of AA and AD remained significantly higher in patients receiving VPIs after adjusting for preexisting hypertension.

Another hypothesis is that medial layer degeneration compromises aortic wall integrity as a result of VEGF pathway inhibition.^26^ VEGF receptors are located on endothelial cells, and signal transduction regulates vascular permeability, cell proliferation, and cell survival.^1,2^ VPIs disrupt downstream signaling pathways, such as the phosphatidylinositol 3-kinase-AKT pathway, resulting in smooth muscle cell apoptosis and imbalanced matrix metalloproteinase expression, which in turn destroys the extracellular matrix.^1,2,26,27,28,29^ This phenomenon may contribute to a loss of aortic wall compliance, strength, and repair, potentially leading to the dilatation of the aortic wall and further development of AA or causing intimal tear and subsequent development of AD.^26,27,28,29,30,31,32^ Our findings indicated a more significant association between VPI use and the risk of AD development. Given that VPI-associated AA and AD may result from the exaggerated pharmacological effects of VPIs, these adverse events can be classified as type A adverse drug reactions (ADRs).^33^ Generally, type A ADRs occur at therapeutic doses in a dose-dependent manner.^33^ Our study revealed a dose-dependent association between VPI use and the risk of AA and AD, which may support the hypothesis.

In this study, we used *ICD-CM* diagnosis codes to define AA and AD, which were validated with a positive predictive rate ranging from 74% to 83%.^19^ Due to the absence of imaging reports in the NHIRD, it is not possible to determine the arterial diameter or the severity of aortic disease only using the diagnosis codes (eTables 3 and 9 in Supplement 1). In Taiwan, physicians typically adhere to the conventional definition of arterial aneurysm, diagnosing aortic aneurysms based on dilation beyond 1.5 times the expected arterial diameter.^34^ As AA is a progressive condition over time, the timing of its diagnosis is influenced by whether imaging examinations are conducted or symptoms appear.^17,34^ This study included patients with cancer who were expected to undergo regular imaging examinations, especially during cancer staging, disease progression, and before receiving the second-line treatments. Therefore, we can infer that the absence of diagnosis codes for AA or AD before exposure to VPI recorded in NHIRD may suggest a low likelihood of the existence of AA and AD before VPI exposure in patients with cancer. Nevertheless, due to the aforementioned reasons, our study only found a nonsignificant increase in the risk of AA with VPI use. Therefore, further prospective longitudinal studies are necessary to confirm the causal association between the use of VPI and the severity of AA and AD.

Under Taiwan’s NHI program, each VPI included in our study has corresponding indications for specific criteria (eTable 1 in Supplement 1). As a result, we conducted a matching process to select controls based on several conditions, including cancer types. Due to the absence of cancer staging information on the index date in the NHIRD, we performed a subgroup analysis focusing on patients with cancer diagnosed at an advanced stage during their initial diagnosis by linking the TCR to NHIRD. This approach aimed to minimize potential exposure misclassification (ie, out-of-pocket use of VPIs) and provide a more homogeneous population for evaluating VPI-associated AA and AD. Although the sample size in the subgroup analysis was relatively small and the results did not reach the threshold for statistical significance, it is worth noting the point estimate of these results align with the results in the main analysis. Further studies are required to confirm the association between VPT exposure and the risk of AA and AD in different types or stages of cancer.

### Limitations

There were some limitations in our study. First, we were unable to adjust covariates that were not available in the NHIRD, such as blood pressure, body mass index, and family history. Second, we could only investigate the 3 oral VPIs that have been covered by the NHI program in Taiwan since before 2012.^21,22^ For the same reason, there was potentially out-of-pocket use of VPIs in both the case and control groups, which could not be identified in the NHIRD. Third, as a retrospective observational study, we were unable to fully account for the presence of genetic aortopathies and connective tissue diseases. While the claims database used in this study may not have accurately captured these patients, we used proxies to help identify patients at baseline with potential connective tissue diseases, including those with a history of Marfan syndrome and those taking medications for inflammatory conditions and autoimmune disorders. Of note, we did not identify any patients in this study with a history of Marfan syndrome. However, other genetic aortopathies were not accounted for. Fourth, we may potentially exclude healthier controls in our study because the controls were selected from patients with inpatient admissions or ED visits. However, this approach was to ensure that the health status and health care utilization of the controls were more comparable to the cases. Finally, we observed a low prevalence of VPIs use from the NHIRD due to restricted coverage by the NHI program, potentially limiting the generalizability of our study results. Future studies could be conducted to include a wider range of populations receiving VPIs, including larger indications and earlier stages of cancer.

## Conclusions

In this case-control study, the use of sorafenib, sunitinib, and pazopanib was associated with a dose-dependent increase in the risk of AA and AD, essentially all of the risk from VPI-associated AD. Health care professionals should closely monitor patients undergoing VPI therapy for the development of AA and AD. Further research is necessary to determine the precise mechanisms and risk factors of VPI-associated AA and AD.

## Notes

Supplement 1.
eMethods.
**eTable 1.** The Reimbursement Criteria for Sorafenib, Sunitinib, and Pazopanib by the National Health Insurance Program in Taiwan **eTable 2.** *ICD-10-CM* and *ICD-9-CM* Codes for Identifying Cancer Diagnoses and Comorbidities **eTable 3.** *ICD-10-CM* and *ICD-9-CM* Codes for Identifying AA and AD Diagnoses **eTable 4.** ATC Codes for the Study Medications **eTable 5.** Socioeconomic Status, Comorbidities, and Concomitant Medications of the Cases With AA and AD and Matched Controls **eTable 6.** Association Between VPI Use and the Risk of AA **eTable 7.** Association Between VPI Use and the Risk of AD **eTable 8.** Association Between VPI Use and the Risk of AA and AD in Patients With Stage IV or Metastatic Cancer **eTable 9.** The Distribution of Locations and Conditions of Aortic Aneurysm

Supplement 2.
Data Sharing Statement
