# Supplementary material for: Vascular Endothelial Growth Factor Inhibitors and the Risk of Aortic Aneurysm and Aortic Dissection
Source: JAMA Netw Open. 2024 Mar 4;7(3):e240940. doi: 10.1001/jamanetworkopen.2024.0940 (PMC10912963; doi:10.1001/jamanetworkopen.2024.0940)
Supplement: Supplement 1. — eMethods. eTable 1. The Reimbursement Criteria for Sorafenib, Sunitinib, and Pazopanib by the National Health Insurance Program in Taiwan eTable 2. ICD-10-CM and ICD-9-CM Codes for Identifying Cancer Diagnoses and Comorbidities eTable 3. ICD-10-CM and ICD-9-CM Codes for Identifying AA and AD Diagnoses eTable 4. ATC Codes for the Study Medications eTable 5. Socioeconomic Status, Comorbidities, and Concomitant Medications of the Cases With AA and AD and Matched Controls eTable 6. Association Between VPI Use and the Risk of AA eTable 7. Association Between VPI Use and the Risk of AD eTable 8. Association Between VPI Use and the Risk of AA and AD in Patients With Stage IV or Metastatic Cancer eTable 9. The Distribution of Locations and Conditions of Aortic Aneurysm [file jamanetwopen-e240940-s001.pdf]

Supplemental Online Content

Wu C, Huang H, Lin S, Wang C, Huang C, Wu I. Vascular endothelial growth factor inhibitors and the risk of aortic aneurysm and aortic dissection. *JAMA Netw Open.* 2024;7(3):e240940. doi:10.1001/jamanetworkopen.2024.0940

eMethods.

eTable 1. The Reimbursement Criteria for Sorafenib, Sunitinib, and Pazopanib by the National Health Insurance Program in Taiwan

eTable 2. *ICD-10-CM* and *ICD-9-CM* Codes for Identifying Cancer Diagnoses and Comorbidities

eTable 3. *ICD-10-CM* and *ICD-9-CM* Codes for Identifying AA and AD Diagnoses

eTable 4. ATC Codes for the Study Medications

eTable 5. Socioeconomic Status, Comorbidities, and Concomitant Medications of the Cases With AA and AD and Matched Controls

eTable 6. Association Between VPI Use and the Risk of AA

eTable 7. Association Between VPI Use and the Risk of AD

eTable 8. Association Between VPI Use and the Risk of AA and AD in Patients With Stage IV or Metastatic Cancer

eTable 9. The Distribution of Locations and Conditions of Aortic Aneurysm

This supplemental material has been provided by the authors to give readers additional information about their work.

© 2024 Wu C et al. *JAMA Network Open.*

eMethods

Data Source

The NHIRD provides patient-level claims data from the National Health Insurance (NHI) program, which includes more than 23 million enrollees (approximately 99.9% of the Taiwanese population).[1] The database also contains de-identified information pertaining to healthcare services, such as insurance enrollment, outpatient and inpatient diagnoses, prescription drug uses, and procedures. The TCR provides information on the initial cancer diagnosis, staging, and treatment. We linked the NHIRD to TCR to identify patients' cancer staging.

Study Design

We chose the nested case-control design because of the following reasons and advantages. First, given the relatively low average incidence of AA and AD in Taiwan, the cohort study design may pose challenges in collecting an adequate number of cases for these rare events. This challenge was particularly significant in this study, as the target population comprised cancer patients, with a limited number of individuals characterized by a relatively short survival period. Second, due to the restricted coverage under Taiwan's NHI program (eTable 1), VPIs are generally prescribed for specific cancer types and advanced cancer stages. Selecting an appropriate active comparator is challenging because of the variations in the treatment regimens across cancers. As a result, this study compared patients exposed to VPIs with those not exposed, allowing unexposed patients to receive alternative anti-cancer drugs. The nested case-control study design allowed us to match the cohort entry date and the index date and to construct a consistent look-back window for cases and matched controls. This approach was intended to assess the association of VPI exposure and risk of AA and AD in the same time window, helping mitigate potential time-related bias, such as immortal time bias, that may occur when comparing VPI exposure and non-exposure in a cohort study design.

VPI Exposure

We adopted the median cumulative duration or dose as the reference point to stratify patients into two groups: those with a cumulative duration/dose less than the median and those equal to or greater than the median. The cumulative duration and dose of VPI exposure were calculated from the cohort entry date to the index date. These cumulative metrics included all

© 2024 Wu C et al. *JAMA Network Open.*

exposed days and were not necessarily consecutive. Additionally, we used the defined daily dose (DDD) as a standardized unit to calculate cumulative doses across the study VPIs. The World Health Organization (WHO) defines the DDD as the assumed average maintenance dose per day for a drug used for its main indication in adults. The WHO has established the DDD as 800 mg for sorafenib, 33 mg for sunitinib, and 800 mg for pazopanib.[2] For example, if a patient took 400 mg of sorafenib daily for a total of 10 days, the cumulative duration and dose would be 10 days and 5 DDDs (0.5 DDD per day*10 days), respectively.

References

1. Hsieh CY, Su CC, Shao SC, et al. Taiwan's National Health Insurance Research Database: past and future. *Clin Epidemiol.* 2019;11:349-358.
2. ATC/DDD Index. *WHO Collaborating Centre for Drug Statistics Methodology.*https://www.whocc.no/atc_ddd_index/. Accessed September 6, 2022.

eTable 1. The reimbursement criteria for sorafenib, sunitinib, and pazopanib by the National Health Insurance program in Taiwan

VEGF pathway inhibitors	Reimbursement criteria
**Sorafenib**	**Renal cancer:**<br>Patients who have failed or are not suitable for treatment with interferon-alpha or interleukin-2.<br>**Liver cancer:**<br>Patients with advanced hepatocellular carcinoma of Child-Pugh A class, characterized by metastasis or inability for surgical resection, and deemed unsuitable for local treatment or experiencing failure of local treatment.
**Sunitinib**	**Renal cancer:**<br>Patients with advanced or metastatic renal cell carcinoma as first-line treatment.<br>**Pancreatic neuroendocrine tumor:**<br>Patients with progressive, unresectable, or metastatic well-differentiated pancreatic neuroendocrine tumors.<br>**Gastrointestinal stromal tumor:**<br>Patients who experience disease progression during treatment with imatinib or develop intolerance to imatinib.
**Pazopanib**	**Renal cancer:**<br>Patients with advanced or metastatic renal cell carcinoma as first-line treatment, pathologically diagnosed as clear cell renal carcinoma.

© 2024 Wu C et al. *JAMA Network Open.*

eTable 2. ICD-10-CM and ICD-9-CM codes for identifying cancer diagnoses and comorbidities

Diseases	ICD-10-CM codes	ICD-9-CM codes
Renal cancer	C64.1, C64.2, C64.9, C65.1, C65.2, C65.9	189.0, 189.1
Hepatic cancer	C22.0, C22.2, C22.3, C22.4, C22.7, C22.8, C22.1, C22.9	155.0, 155.2, 155.1
Gastrointestinal cancer	C16.0–C16.9, C17.0–C17.9, C48.0, C26.0, C26.9, C47.4, C49.4, D49.0	151.0–151.9, 152.0–152.9, 158.0, 159.0, 159.8, 159.9, 171.5, 239.0
Pancreatic cancer	C7A.1, C7A.8, C7B.8, C25.0–C25.9	199.0, 199.1, 157.0–157.9
Heart failure	I11.0, I13.0, I13.2, I25.5, I42.0, I42.5, I42.6, I42.7, I42.8, I42.9, I43, I50, P29.0	398.91, 402.01, 402.11, 402.91, 404.01, 404.03, 404.11, 404.13, 404.91, 404.93, 425.4, 425.5, 425.6, 425.7, 425.8, 425.9, 428
Cerebrovascular disease	I60–I69, G45, G46, H34.0, H34.1, H34.2	430–438, 362.34
Coronary artery disease	I20–I25	410–414
Peripheral arterial disease	I70.1–I70.9, I72, I73, I77, I79.8	440.1–440.9, 442, 443
Diabetes mellitus	E08–E13	250
Hypertension	I10–I16	401–405
Lipid disorder	E78	272
Atrial fibrillation	I48.0, I48.1, I48.2, I48.91	427.31
Aortic valve disease	I06, I08.0, I08.2, I08.3, I35, Q23.0, Q23.8, Q23.9	395, 396, 424.1, 746.3
Bicuspid aortic valve	Q23.1	746.4
Obstructive sleep apnea	G47.3	780.51, 780.53, 780.57
Chronic obstructive pulmonary disease	J44	496
Asthma	J45	493
Chronic kidney disease	N18	585
Tobacco use	F17.21, F17.22, F17.29, O99.33, T65.2, Z71.6, Z72.0, Z87.891	305.1, 989.84, V15.82
Obesity	E66	278
Marfan syndrome	Q87.4	759.82
Cardiovascular syphilis	A52.0	093
Schizophrenia	F20	295
Seizure disorders	G40	345

© 2024 Wu C et al. *JAMA Network Open.*

eTable 3. ICD-10-CM and ICD-9-CM codes for identifying AA and AD diagnoses

Diseases	ICD-10-CM codes	ICD-9-CM codes
Aortic aneurysm and dissection	I71	441
Dissection of aorta	I71.0	441.0
Thoracic aortic aneurysm, ruptured	I71.1	441.1
Thoracic aortic aneurysm, without rupture	I71.2	441.2
Abdominal aortic aneurysm, ruptured	I71.3	441.3
Abdominal aortic aneurysm, without rupture	I71.4	441.4
Thoracoabdominal aortic aneurysm, ruptured	I71.5	441.6
Thoracoabdominal aortic aneurysm, without rupture	I71.6	441.7
Aortic aneurysm of unspecified site, ruptured	I71.8	441.5
Aortic aneurysm of unspecified site, without rupture	I71.9	441.9
Aneurysm of aorta in diseases classified elsewhere	I79.0	nil
Aortitis in diseases classified elsewhere	I79.1	nil

AA: aortic aneurysm; AD; aortic dissection

© 2024 Wu C et al. *JAMA Network Open.*

eTable 4. ATC codes for the study medications

Medications	ATC codes
Sorafenib	L01EX02
Pazopanib	L01EX03
Sunitinib	L01EX01, L01XE04
NSAIDs	M01A, M01B, N02BA
Immunosuppressants	L04
Systemic corticosteroids	H02A, H02BX
DMARDs	A07EC, L01BA, P01BA
Statins	C10AA, C10BA01–C10BA09, C10BX
Fibrates	C10AB
ACEIs	C09AA, C09B
ARBs	C09C, C09D
Beta-blockers	C07
CCBs	C08
Oral beta-2 agonists	R03C
Nitrates	C01DA
Peripheral vasodilators	C04A
Oral anticoagulants	B01AA, B01AE07, B01AF
Antiplatelet drugs	B01AC, B01AC04, B01AC05, B01AC06, B01AC07, B01AC22, B01AC23, B01AC24, B01AC56
Antiarrhythmic agents	C01B
Digoxin	C01AA, C03C
Insulin	A10AB, A10AC, A10AD, A10AE
Oral hypoglycemic agents	A10B
Antidepressants	N06A
Benzodiazepines	N05AH, N05BA, N05CD
Drugs against Parkinson disease	N04
Antipsychotics	N05AA, N05AB, N05AC, N05AD, N05AE, N05AF, N05AG, N05AK, N05AL, N05AN, N05AX
Antiseizure drugs	N03
Hydroxyzine	N05BB01
Fluoroquinolones	J01MA, J01MB

ACEI: angiotensin-converting enzyme inhibitor; ARB: angiotensin II receptor blocker; ATC: Anatomical Therapeutic Chemical; CCB: calcium channel blocker; DMARD: disease-modifying antirheumatic drug; NSAID: nonsteroidal anti-inflammatory drug.

© 2024 Wu C et al. *JAMA Network Open.*

eTable 5. Socioeconomic status, comorbidities, and concomitant medications of the cases with AA and AD and matched controls

	Cases<br><br>*N* = 1,461	Controls<br><br>*N* = 7,198	*P*
**Socioeconomic status**			
**Insurance fees**			
**<21,000 NTD**	481 (32.9)	2,387 (33.2)	0.1239
**21,000–23,000 NTD**	459 (31.4)	2,360 (32.8)	
**>24,000 NTD**	515 (35.3)	2,440 (33.9)	
**Unknown**	6 (0.4)	11 (0.2)	
**Comorbidities**			
**CCI, mean (SD)**	3.67 (3.1)	3.37 (3.0)	0.0002
**Risk factors**			
**Heart failure**	155 (10.6)	472 (6.6)	<0.0001
**Cerebrovascular disease**	226 (15.5)	877 (12.2)	0.0006
**Coronary artery disease**	332 (22.7)	1,266 (17.6)	<0.0001
**Peripheral arterial disease**	66 (4.5)	172 (2.4)	<0.0001
**Diabetes mellitus**	324 (22.2)	2,041 (28.4)	<0.0001
**Hypertension**	930 (63.7)	3,963 (55.1)	<0.0001
**Lipid disorder**	330 (22.6)	1,519 (21.1)	0.1539
**Atrial fibrillation**	14 (1.0)	87 (1.2)	0.3853
**Aortic valve disease**	65 (4.5)	108 (1.5)	<0.0001
**Obstructive sleep apnea**	8 (0.6)	27 (0.4)	0.3343
**Chronic obstructive pulmonary disease**	144 (9.9)	458 (6.4)	<0.0001
**Asthma**	95 (6.5)	326 (4.5)	0.0013
**Chronic kidney disease**	168 (11.5)	691 (9.6)	0.0301
**Tobacco use**	10 (0.7)	16 (0.2)	0.0042
**Obesity**	4 (0.3)	11 (0.2)	0.2987
**Seizure disorder**	14 (1.0)	53 (0.7)	0.3472
**Concomitant medications**			
**Within 365 days prior to the cohort entry date**			
**NSAIDs**	73 (5.0)	397 (5.5)	0.4213
**Immunosuppressants**	7 (0.5)	26 (0.4)	0.4809
**Systemic corticosteroids**	100 (6.8)	401 (5.6)	0.0513
**DMARDs**	10 (0.7)	44 (0.6)	0.7140
**Statins**	96 (6.6)	410 (5.7)	0.2182
**Fibrates**	32 (2.2)	138 (1.9)	0.5093
**ACEIs**	82 (5.6)	358 (5.0)	0.3832
**ARBs**	142 (9.7)	652 (9.1)	0.4050
**Beta-blockers**	141 (9.7)	647 (9.0)	0.4039
**CCBs**	158 (10.8)	713 (9.9)	0.3002
**Oral beta-2 agonists**	54 (3.7)	248 (3.5)	0.6460
**Nitrates**	92 (6.3)	350 (4.9)	0.0224
**Peripheral vasodilators**	92 (6.3)	411 (5.7)	0.4229
**Oral anticoagulants**	24 (1.6)	100 (1.4)	0.4421
**Antiplatelet drugs**	139 (9.5)	602 (8.4)	0.1361
**Antiarrhythmic agents**	60 (4.1)	236 (3.3)	0.1018
**Digoxin**	119 (8.2)	533 (7.4)	0.3387
**Insulin**	33 (2.3)	229 (3.2)	0.0679
**Oral hypoglycemic agents**	68 (4.7)	432 (6.0)	0.0362
**Antidepressants**	97 (6.6)	421 (5.9)	0.2160
**Benzodiazepines**	145 (9.9)	659 (9.2)	0.3203
**Drugs against Parkinson disease**	26 (1.8)	103 (1.4)	0.3126
**Antipsychotics**	47 (3.2)	226 (3.14)	0.8606
**Antiseizure drugs**	75 (5.1)	341 (4.7)	0.5099
**Hydroxyzine**	19 (1.3)	108 (1.5)	0.5770

© 2024 Wu C et al. *JAMA Network Open.*

**Fluoroquinolones**	89 (6.1)	420 (5.8)	0.7204
**Within 100 days prior to the index date**			
**NSAIDs**	8 (0.6)	52 (0.7)	0.4810
**Immunosuppressants**	4 (0.3)	11 (0.2)	0.2987
**Systemic corticosteroids**	45 (3.1)	223 (3.1)	0.9096
**DMARDs**	4 (0.3)	20 (0.3)	1.0000
**Statins**	44 (3.0)	138 (1.9)	0.0066
**Fibrates**	5 (0.3)	32 (0.4)	0.6048
**ACEIs**	33 (2.3)	125 (1.7)	0.1830
**ARBs**	43 (2.9)	213 (3.0)	0.9768
**Beta-blockers**	61 (4.2)	287 (4.0)	0.7054
**CCBs**	38 (2.6)	200 (2.8)	0.7341
**Nitrates**	38 (2.6)	136 (1.9)	0.0629
**Peripheral vasodilators**	17 (1.2)	94 (1.3)	0.6709
**Oral beta-2 agonists**	26 (1.8)	137 (1.9)	0.7202
**Oral anticoagulants**	16 (1.1)	60 (0.8)	0.3036
**Antiplatelet drugs**	40 (2.7)	199 (2.8)	0.9758
**Antiarrhythmic agents**	20 (1.4)	202 (2.8)	0.0013
**Digoxin**	65 (4.5)	453 (6.3)	0.0060
**Insulin**	28 (1.9)	224 (3.1)	0.0128
**Oral hypoglycemic agents**	21 (1.4)	149 (2.1)	0.1086
**Antidepressants**	29 (2.0)	129 (1.8)	0.5924
**Benzodiazepines**	42 (2.9)	232 (3.2)	0.5376
**Drugs against Parkinson disease**	6 (0.4)	34 (0.5)	0.7773
**Antipsychotics**	35 (2.4)	177 (2.5)	0.9007
**Antiseizure drugs**	32 (2.2)	133 (1.9)	0.3413
**Hydroxyzine**	11 (0.8)	50 (0.7)	0.7739
**Fluoroquinolones**	60 (4.1)	278 (3.9)	0.5900

Abbreviations: ACEI: angiotensin-converting enzyme inhibitor; ARB: angiotensin II receptor blocker; CCB: calcium channel blocker; CCI: Charlson Comorbidity Index; DMARD: disease-modifying antirheumatic drug; IQR: interquartile range; NSAID: nonsteroidal anti-inflammatory drug; NTD: New Taiwan Dollars; SD: standard deviation.
Note: Data are presented as the number of patients (%) unless indicated otherwise.

© 2024 Wu C et al. *JAMA Network Open.*

eTable 6. Association between VPI[a] use and the risk of AA

	Cases *N* = 443	Controls *N* = 2,197	Unadjusted OR (95% CI)	Adjusted OR[e] (95% CI)
**Risk windows of VPI exposure (days before the index date[b])**				
≤ 100 days	18 (24.0)	57 (76.0)	1.61 (0.93-2.77)	1.68 (0.96-2.93)
101-365 days	0 (0.0)	10 (100.0)	—	—
> 365 days	4 (25.0)	12 (75.0)	1.72 (0.53-5.58)	1.830 (0.57-5.92)
Non-exposure	933 (16.8)	4607 (83.2)	*Reference*	*Reference*
**VPI cumulative days[c]**				
< 68 days	9 (18.0)	41 (82.0)	1.11 (0.54-2.31)	1.14 (0.54-2.39)
≥ 68 days	13 (25.5)	38 (74.5)	1.74 (0.91-3.32)	1.89 (0.98-3.65)
Non-exposure	933 (16.8)	4607 (83.2)	*Reference*	*Reference*
**VPI cumulative doses[c]**				
< 61 DDDs[d]	10 (19.6)	41 (80.4)	1.23 (0.61-2.50)	1.23 (0.60-2.51)
≥ 61 DDDs[d]	12 (24.0)	38 (76.0)	1.61 (0.82-3.14)	1.81 (0.92-3.57)
Non-exposure	933 (16.8)	4607 (83.2)	*Reference*	*Reference*

AA: aortic aneurysm; CI: confidence interval; DDD: defined daily dose; OR: odds ratio; VPI: vascular endothelial growth factor pathway inhibitor.

Note: Data are presented as the number of patients (row %).

[a]Sorafenib, sunitinib, and pazopanib.

[b]The date of the first AA event.

[c]Between the cohort entry date (i.e., the date of the initial cancer diagnosis) and the index date (i.e., the date of the first AA/AD event).

[d]According to the definitions of the World Health Organization, the DDDs were 800 mg for sorafenib, 33 mg for sunitinib, and 800 mg for pazopanib.

[e]Adjusted ORs were obtained using multivariable conditional logistic regression models. Baseline conditions with statistically significant OR differences between cases and controls were adjusted as covariates, including socioeconomic status, heart failure, cerebrovascular disease, coronary artery disease, peripheral arterial disease, diabetes, hypertension, aortic valve disease, chronic obstructive pulmonary disease, chronic kidney disease, seizure, concomitant medication use within 365 days before the cohort entry date (insulin), and concomitant medication use within 100 days before the index date (antiarrhythmic agents, digoxin, insulin, hydroxyzine, and anticonvulsants).

© 2024 Wu C et al. *JAMA Network Open.*

eTable 7. Association between VPI[a] use and the risk of AD

	Cases *N* = 443	Controls *N* = 2,197	Unadjusted OR (95% CI)	Adjusted OR[e] (95% CI)
**Risk windows of VPI exposure (days before the index date[b])**				
≤ 100 days	17 (33.3)	34 (66.7)	2.83 (1.51-5.31)	2.93 (1.51-5.70)
101-365 days	4 (40.0)	6 (60.0)	3.52 (0.93-13.30)	4.10 (1.07-15.70)
> 365 days	3 (33.3)	6 (66.7)	2.67 (0.67-10.72)	2.88 (0.70-11.84)
Non-exposure	419 (16.3)	2151 (83.7)	*Reference*	*Reference*
**VPI cumulative days[c]**				
< 68 days	10 (27.8)	26 (72.2)	2.12 (0.99-4.53)	2.27 (1.02-5.05)
≥ 68 days	14 (41.2)	20 (58.8)	3.94 (1.92-8.08)	4.14 (1.95-8.78)
Non-exposure	419 (16.3)	2151 (83.7)	*Reference*	*Reference*
**VPI cumulative doses[c]**				
< 61 DDDs[d]	10 (28.6)	25 (71.4)	2.18(1.02-4.70)	2.16 (0.97-4.80)
≥ 61 DDDs[d]	14 (40.0)	21 (60.0)	3.81 (1.86-7.81)	4.35 (2.05-9.20)
Non-exposure	419 (16.3)	2151 (83.7)	*Reference*	*Reference*

AD: aortic dissection; CI: confidence interval; DDD: defined daily dose; OR: odds ratio; VPI: vascular endothelial growth factor pathway inhibitor.
Note: Data are presented as the number of patients (row %).
[a]Sorafenib, sunitinib, and pazopanib.
[b]The date of the first AD event.
[c]Between the cohort entry date (i.e., the date of the initial cancer diagnosis) and the index date (i.e., the date of the first AA/AD event).
[d]According to the definitions of the World Health Organization, the DDDs were 800 mg for sorafenib, 33 mg for sunitinib, and 800 mg for pazopanib.
[e]Adjusted ORs were obtained using multivariable conditional logistic regression models. Baseline conditions with statistically significant OR differences between cases and controls were adjusted as covariates, including heart failure, cerebrovascular disease, coronary artery disease, peripheral arterial disease, diabetes, hypertension, aortic valve disease, chronic obstructive pulmonary disease, asthma, chronic kidney disease, tobacco use, Charlson Comorbidity Index, concomitant medication use within 365 days before the cohort entry date (oral hypoglycemic agents, nitrates, and anticonvulsants), and concomitant medication use within 100 days before the index date (statins, antiarrhythmic agents, digoxin, and insulin).

© 2024 Wu C et al. *JAMA Network Open.*

eTable 8. Association between VPI[a] use and the risk of AA and AD in patients with stage IV or metastatic cancer

	Cases *N* = 56	Controls *N* = 258	Unadjusted OR (95% CI)	Adjusted OR[e] (95% CI)
**Risk windows of VPI exposure (days before the index date[b])**				
≤ 100 days	4 (13.8)	25 (86.2)	2.00 (0.18-22.06)	—
101-365 days	0 (0.00)	1(100.0)	—	—
> 365 days	0 (0.00)	3 (100.0)	—	—
Non-exposure	52 (18.5)	229 (81.5)	*Reference*	*Reference*
**VPI cumulative days[c]**				
< 68 days	0 (0.0)	18 (100.0)	—	—
≥ 68 days	4 (26.7)	11 (73.3)	—	—
Non-exposure	52 (18.5)	229 (81.5)	*Reference*	*Reference*
**VPI cumulative doses[c]**				
< 61 DDDs[d]	0 (0.0)	18 (100.0)	—	—
≥ 61 DDDs[d]	4 (26.7)	11 (73.3)	—	—
Non-exposure	52 (18.5)	229 (81.5)	*Reference*	*Reference*

AA: aortic aneurysm; AD: aortic dissection; CI: confidence interval; DDD: defined daily dose; OR: odds ratio; VPI: vascular endothelial growth factor pathway inhibitor.
Note: Data are presented as the number of patients (row %).
[a]Sorafenib, sunitinib, and pazopanib.
[b]The date of the first AA and AD event.
[c]Between the cohort entry date (i.e., the date of the initial cancer diagnosis) and the index date (i.e., the date of the first AA/AD event).
[d]According to the definitions of the World Health Organization, the DDDs were 800 mg for sorafenib, 33 mg for sunitinib, and 800 mg for pazopanib.
[e]Adjusted ORs were obtained using multivariable conditional logistic regression models. Baseline conditions with statistically significant OR differences between cases and controls were adjusted as covariates, including cerebrovascular disease, coronary artery disease, peripheral arterial disease, diabetes, hypertension, aortic valve disease, chronic obstructive pulmonary disease, asthma, chronic kidney disease, tobacco use, Charlson Comorbidity Index, concomitant medication use within 365 days before the cohort entry date (oral hypoglycemic agents and nitrates), and concomitant medication use within 100 days before the index date (statins, antiarrhythmic agents, digoxin, and insulin).

© 2024 Wu C et al. *JAMA Network Open.*

eTable 9. The distribution of locations and conditions of aortic aneurysm

**Aortic aneurysm**	N=1,340<br>n (%)
Thoracic aortic aneurysm, ruptured	35 (2.61)
Thoracic aortic aneurysm, without rupture	224 (16.72)
Abdominal aortic aneurysm, ruptured	46 (3.43)
Abdominal aortic aneurysm, without rupture	588 (43.88)
Thoracoabdominal aortic aneurysm, ruptured	3 (0.22)
Thoracoabdominal aortic aneurysm, without rupture	14 (1.04)
Aortic aneurysm of unspecified site, ruptured	13 (0.97)
Aortic aneurysm of unspecified site, without rupture	120 (8.96)
Aneurysm of aorta in diseases classified elsewhere	297 (22.16)

Note: Data are presented as the number of diagnoses (%). One patients may have multiple diagnoses.

© 2024 Wu C et al. *JAMA Network Open.*
