# Supplementary material for: Vascular Endothelial Growth Factor Inhibitors and the Risk of Aortic Aneurysm and Aortic Dissection
Source: JAMA Netw Open. 2024 Mar 4;7(3):e240940. doi: 10.1001/jamanetworkopen.2024.0940 (PMC10912963; doi:10.1001/jamanetworkopen.2024.0940)
Supplement: Supplement 2. — Data Sharing Statement [file jamanetwopen-e240940-s002.pdf]

# Data Sharing Statement

Wu. Vascular Endothelial Growth Factor Inhibitors and the Risk of Aortic Aneurysm and Aortic Dissection. *JAMA Netw Open*. Published March 04, 2024.
doi:10.1001/jamanetworkopen.2024.0940

**Data**
**Data available:** No

**Additional Information**
**Explanation for why data not available:** The datasets analyzed during the current study are not publicly available due to legal restrictions by Taiwan's policy. Data are available from the corresponding author on reasonable request and with permission from the Health and Welfare Data Science Center, Ministry of Health and Welfare in Taiwan.
